# Factors predicting surgical site infection after posterior lumbar surgery: A multicenter retrospective study: Retraction

**DOI:** 10.1097/MD.0000000000007102

**Published:** 2017-05-26

In the article, “Factors predicting surgical site infection after posterior lumbar surgery: A multicenter retrospective study”,^[1]^ which appeared in Volume 96, Issue 5 of *Medicine*, in Table 2, some of the confidence intervals are incorrect because the wrong mode was selected in the statistical operations. Due to this incorrect data, this article is being retracted.
